# The role of ultrasonography in diagnosing acute closed volar plate injury of proximal interphalangeal joint

**DOI:** 10.1186/s12880-023-01079-2

**Published:** 2023-09-04

**Authors:** Tiezheng Wang, Fei Guo, Hengtao Qi, Liyuan Cui, Lihua Liu, Shougang Bao, Jianbo Teng

**Affiliations:** 1 Department of Ultrasound, Shandong Provincial Hospital Affiliated to Shandong First Medical University, No.324, Jingwu Road, Jinan, 250021 Shandong China; 2 Department of Ultrasound, Hospital of Weihai Municipal Hospital, Cheeloo College of Medicine, Shandong University, Weihai, 264200 China

**Keywords:** Ultrasonography, Proximal interphalangeal joint, Volar plate

## Abstract

**Background:**

Acute closed volar plate injury of the proximal interphalangeal joint (PIP) is a common hand injury. In the past, there were few objective evaluation imaging methods for the degree of volar plate injury. The purpose of this study was to investigate the role of high frequency ultrasonography in diagnosing volar plate injury, and to explore whether ultrasound can provide a beneficial guidance to clinical decision-making and appropriate treatment adopting through accurate US classification of volar plate injury.

**Methods:**

From May 2019 to may 2022, 41 patients diagnosed with volar plate injury were included in this study. All patients underwent ultrasonography and X-ray examinations. The sonographic features were analyzed. A new kind of classification of volar plate injury based on ultrasonography findings was described.

**Results:**

Either an injury of volar plate or an avulsion fracture of middle phalangeal base was identified clearly on ultrasonography, according to which volar plate injury could be divided into three types: A, B and C. Type A, avulsion fracture of the middle phalangeal base without volar plate rupture; Type B, full thickness tear of the volar plate without avulsion fracture; Type C, partial thickness tear of the volar plate. The average thickness of the three types of injured volar plate measured by ultrasound was 0.33 ± 0.05 cm, and the average thickness of the volar plate at the same site of the corresponding finger on the contralateral side was 0.22 ± 0.03 cm. There was significant difference between the two group (*t* = 11.823, *p* = 1.2476 *10^(-14)).

**Conclusions:**

High frequency ultrasonography could be a reliable, accurate, convenient and non-radioactive diagnostic imaging technique in the evaluation of acute closed volar plate injury of PIP. And ultrasound could provide a beneficial guidance to clinical decision-making and appropriate treatment adopting through accurate US classification.

## Background

Closed volar plate injury of the proximal interphalangeal joint (PIP) is a common hand injury, which is often accompanied by dorsal dislocation of PIP, caused by hyperextension and rotation violence. In the early stage, volar plate injury presents with instability of joint. If combined with collateral ligament injury, instability of joint flexion and lateral deviation will both occur, which may become an obstacle to the reduction of joint dislocation [1–3]. Although clinicians can make the diagnosis of acute closed volar plate injury according to the symptoms and signs, there are few objective evaluation imaging methods for the degree of acute closed volar plate injury [4–6]. The purpose of this study was to observe the clinical application and usage of high frequency ultrasonography in diagnosing acute closed volar plate injury of PIP, and to provide objective basis for clinical treatment.

## Methods

Forty-one patients each with an acute closed volar plate injury of PIP (age range: 10–55 years; mean age: 33.6 years) referred to the orthopaedic department of our hospital from May 2019 to may 2022 were included into this study. The study protocol was approved by the ethics committee of Provincial Hospital Affiliated to Shandong First Medical University (NO.2021-056). All studies to have been performed in accordance with the ethical standards in the 2002 Declaration of Helsinki and all methods were performed in accordance with the relevant guidelines and regulations. All participants provided written informed consent. All 41 patients were closed injury. Duration of symptoms varied from 1 day to 2 weeks reported by the patients. Detailed clinical data was summarized in Table 1.

**Table 1:** The detailed clinical profiles for closed volar plate injury

Patient no.	Finger affected	Duration of symptoms
1	Left index finger	Four days
2	Right ring finger	14 days
3	Right index finger	Two days
4	Right ring finger	Five days
5	Right ring finger	One day
6	Right middle finger	Five days
7	Right index finger	Three days
8	Right little finger	Four days
9	Right index finger	Seven days
10	Right ring finger	Seven days
11	Right middle finger	14 days
12	Right middle finger	Four days
13	Left middle finger	Two days
14	Right little finger	Four days
15	Right index finger	Seven days
16	Right middle finger	Five days
17	Right index finger	Three days
18	Right index finger	14 days
19	Right index finger	Five days
20	Right ring finger	Two days
21	Left index finger	One day
22	Right middle finger	14 days
23	Right little finger	Four days
24	Right middle finger	Three days
25	Right little finger	One day
26	Right middle finger	Five days
27	Right index finger	Seven days
28	Right index finger	Three days
29	Left little finger	14 days
30	Right index finger	Seven days
31	Left ring finger	Four days
32	Right index finger	Four days
33	Left index finger	One day
34	Right ring finger	Seven days
35	Left index finger	Four days
36	Right middle finger	One day
37	Right little finger	Three days
38	Right middle finger	Four days
39	Right index finger	Four days
40	Right middle finger	One day
41	Right middle finger	Two days

All patients underwent high-frequency ultrasonography and X-ray examination. All the ultrasound and X-ray examinations were performed by experienced musculoskeletal ultrasound or imaging experts with > 10 years of experience in musculoskeletal ultrasound or X-ray examination blind to X-ray or ultrasound data.

A Philips EPIQ 7 ultrasound system (Washington, The USA) and a Canon i800 ultrasound system (TOCHIGI, JAPAN) with a 18 MHz and a 24 MHz broadband linear array probe was used in ultrasonography examination. During the examination, a thick layer of gel or a ultrasound solid gel pad (Chao ji, SN1002) was applied between the transducer and the skin for better observation result. A musculoskeletal preset condition was used in all examinations. Both a static and a dynamic ultrasonography scan with longitudinal (in the central sagittal plane of the digits to display both the profile of the VP and the flexor tendons) and axial images were performed on the injured finger focusing of the volar plate insertion site of the PIP. The static examination was carried out with the injured finger in neutral position, while the dynamic examination was performed by intended active or passive moving PIP and observing the real-time sonograms of the volar plate. A comparison examination of the contralateral healthy finger was carried out for the purpose of control. For patients with complete rupture of the volar plate, the disruption distance between the proximal retracted volar plate stump and the base of the middle phalanx was measured and recorded. The collateral ligament of the PIP was examined to observe its continuity.

Ultrasonography images of the 41 cases in PACS were all assessed by two musculoskeletal radiologists blinded to the clinical diagnosis independently, with more than 15 years of imaging diagnosing experience. Reference to original imaging reports was shielded to avoid bias.

Besides of observing the volar plate, base of the middle phalanx was carefully checked during the ultrasonography examination, as well as on X-ray images to exclude fracture. The ultrasonography and X-ray results of each case were summarized in Tables 2 and 3.

**Table 2:** The image examination results of acute closed volar plate injury (radiologist A)

	US (41 cases)	X-ray(41 cases)
Avulsion fracture without volar plate rupture	13	13
Full thickness tear of volar plate without fracture	11	-
Partial thickness tear of volar plate	17	-

**Table 3:** The image examination results of acute closed volar plate injury (radiologist B)

	US (41 cases)	X-ray(41 cases)
Avulsion fracture without volar plate rupture	13	13
Full thickness tear of volar plate without fracture	12	-
Partial thickness tear of volar plate	16	-

For evaluation of the closed volar plate injury, a new ultrasonography classification method was proposed in this study according to the type and location of the injury (volar plate injury or fracture of the middle phalanx): Type A, avulsion fracture without volar plate rupture. Type B, full thickness tear of volar plate without fracture. Type C, partial thickness tear of volar plate.

The SPSS program (version 25.0, SPSS, Chicago, IL, USA) was used for statistical analysis. Kappa test was adopted to evaluate the inter-rater reliability for the original classifications. Paired t-test was used to find statistical difference of the average measured diameter between acute closed volar plate injury and contralateral fingers.

## Results

**Fig. 1:**
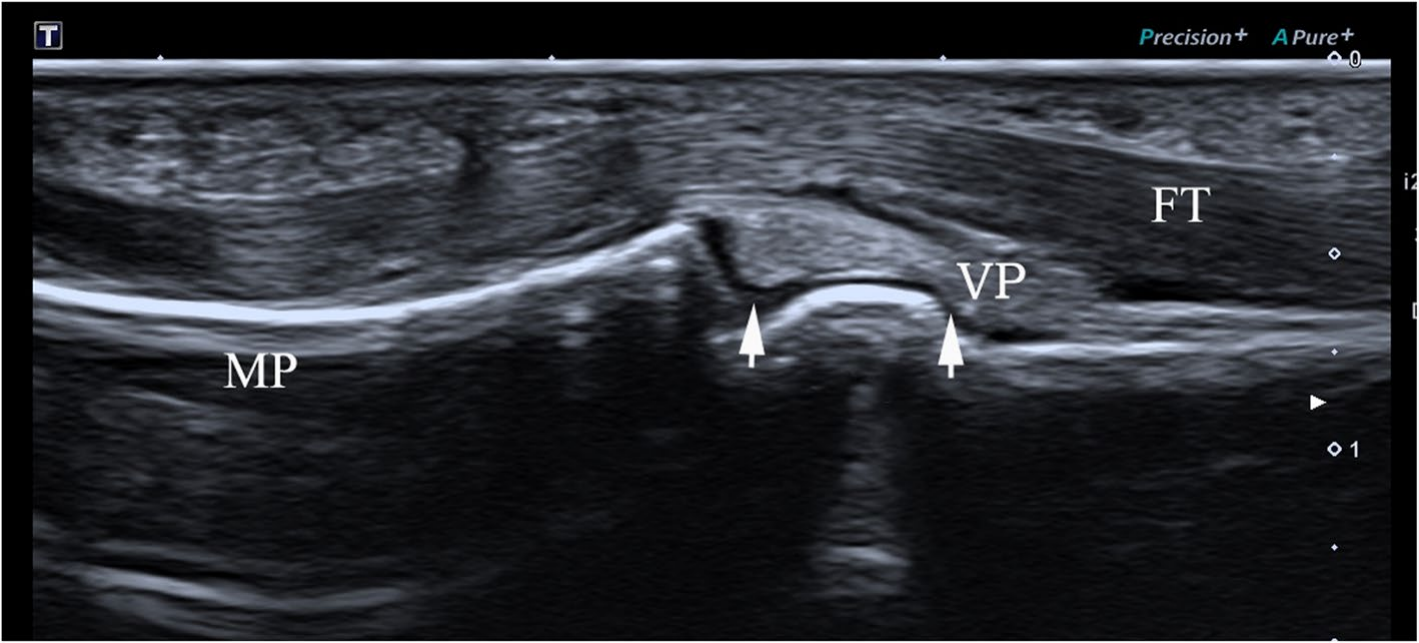
High frequency ultrasonography image of normal volar plate at PIP of a 30-year-old male in longitudinal plane(arrow). VP: volar plate; FT: flexor tendon; MP: middle phalanx

Normal volar plate at the PIP of the finger is located between the flexor tendon and the proximal phalangeal head, which is tightly attached to the phalanx. It appeares irregular hyperecho in longitudinal plane on ultrasonography. As shown in Fig. 1, the proximal end of volar plate is attached to the proximal phalangeal head, and the distal end is attached to the middle phalangeal base. When the joint moves, the sliding of the volar plate could be seen.

**Fig. 2:**
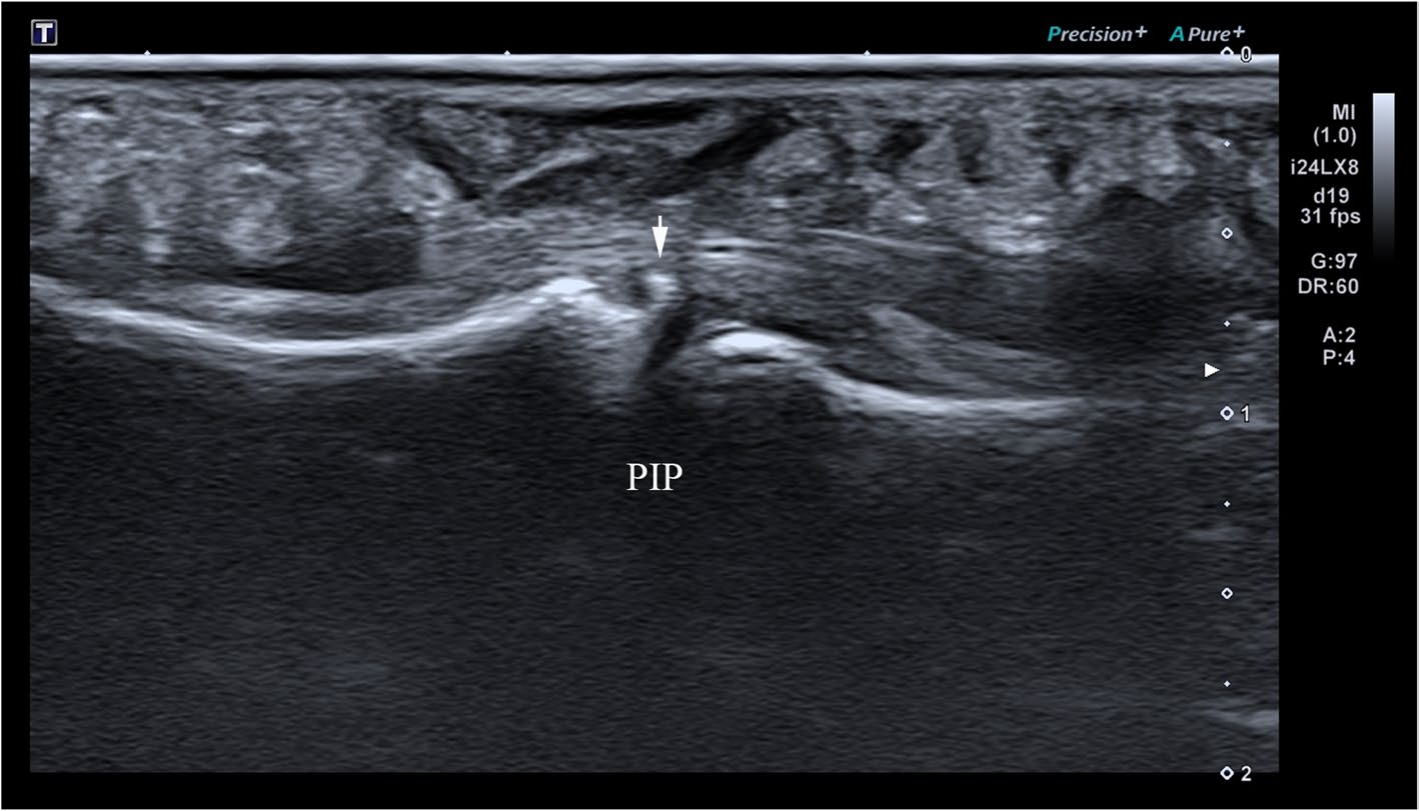
Type A (avulsion fracture without volar plate rupture). High frequency ultrasonography image of type A volar plate injury of a 21-year-old female in longitudinal plane. The arrow shows the avulsion bony fragment of the middle phalangeal base. PIP: proximal interphalangeal joint

**Fig. 3:**
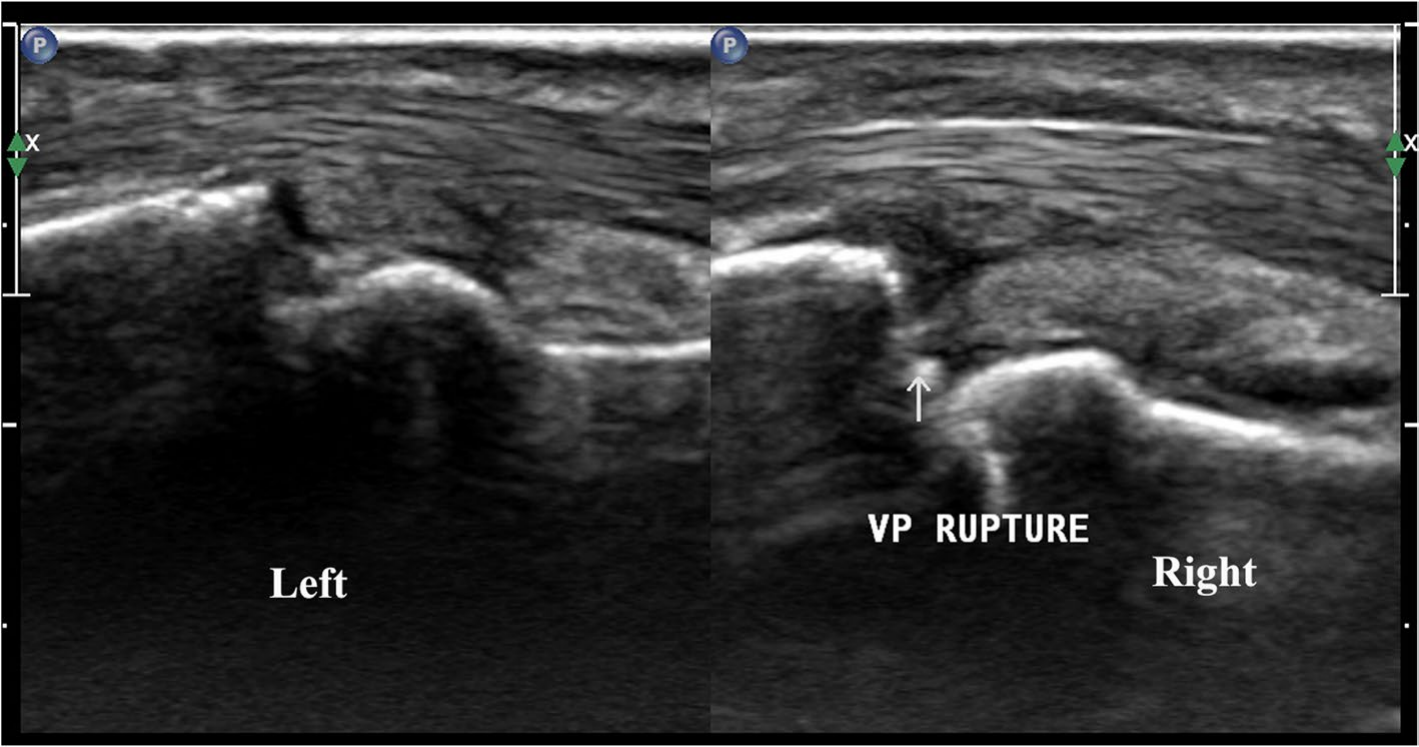
Type B (full thickness tear of volar plate without fracture). A 35-year-old male with type B volar plate injury. The arrow shows the disruption of the volar plate at the level of PIP with retraction of the proximal volar plate stump but no fracture fragments of the middle phalangeal base, and the left side is his normal contralateral volar plate

**Fig. 4:**
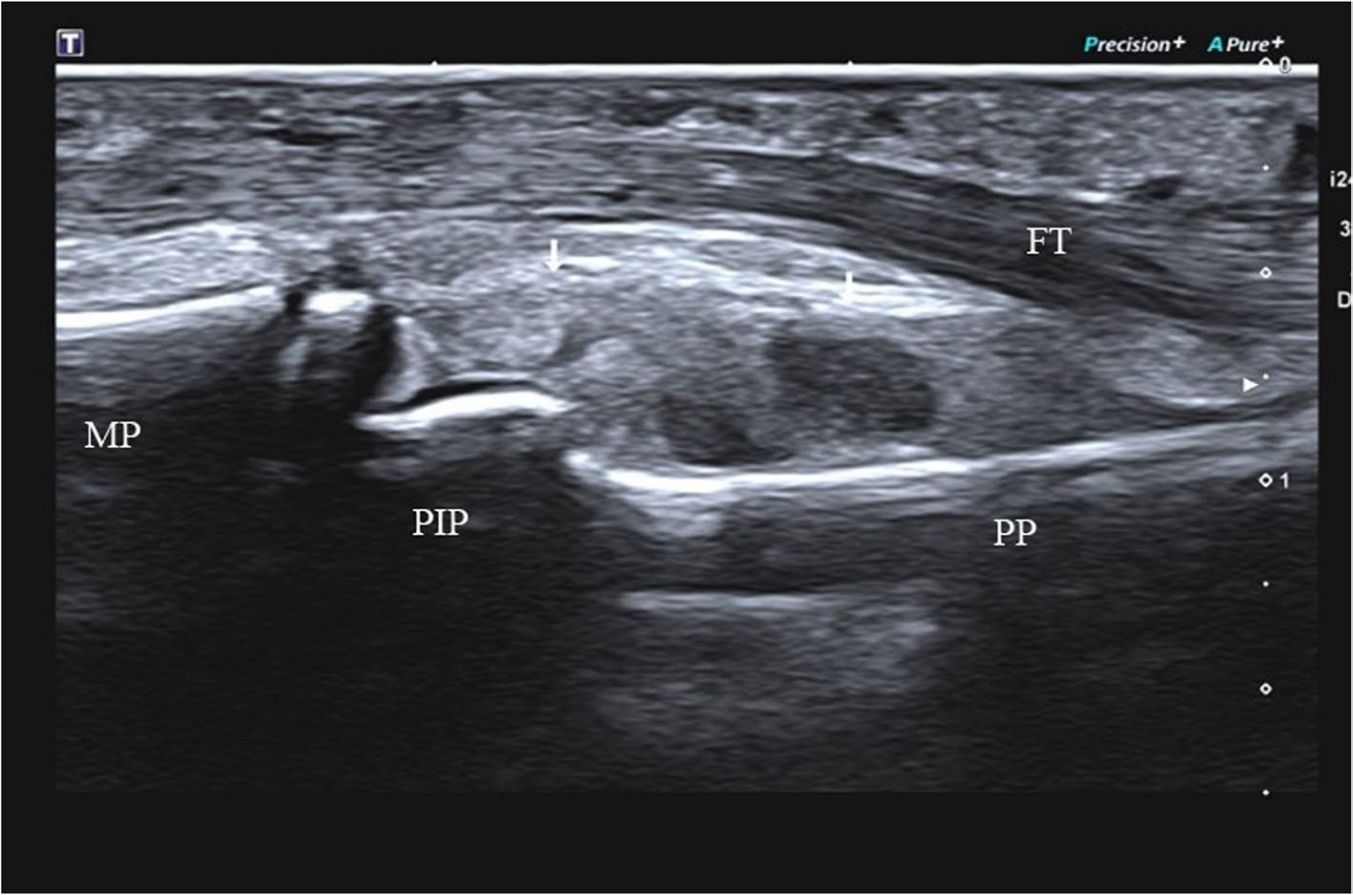
Type C (partial thickness tear of volar plate). High frequency ultrasonography image of type C volar plate injury of a 10-year-old boy in longitudinal plane. The ultrasonography showed the thicker and hypoechoic volar plate which was still integrate in longitudinal plane(arrow). MP: middle phalanx; PP: proximal phalanx; PIP: proximal interphalangeal joint; FT: flexor tendon

High frequency ultrasound can clearly demonstrate the location, degree and whether there is avulsion fracture in patients with acute closed volar plate injury, according to which we can divide them into three types: Type A: avulsion fracture of the middle phalangeal base without volar plate rupture. High frequency ultrasound demonstrated the hyperechoic avulsion fracture of the middle phalangeal base and the thicker volar plate with the hyperechoic avulsion fracture fragment at the distal margin (Fig. 2); the distal portion showed immobility during either active or passive movements of the PIP. Type B: full thickness tear of the volar plate attachment of the middle phalangeal base without avulsion fracture. High frequency ultrasound showed the complete disruption of the volar plate with retraction of the proximal volar plate stump without avulsion fracture fragments of the middle phalangeal base (Fig. 3); volar plate showed immobility in the volar-dorsal direction during either active or passive movements of the PIP. Type C: partial thickness tear of the volar plate. In longitudinal plane, high frequency ultrasound demonstrated the hypoechoic and thicker volar plate which was still integrate (Fig. 4). Real-time movement of the volar plate could be found during both active and passive movements of the PIP. The the inter-rater reliability for the classifications was high between two radiologists (κ = 0.963).

The average thickness of the three types of closed volar plate injury of the PIP measured by ultrasound was 0.33 ± 0.05 cm, and the average thickness of the volar plate at the same site of the corresponding finger on the contralateral side was 0.22 ± 0.03 cm. There was significant difference between the two group (*t* = 11.823, *p* = 1.2476 *10^(-14)). And the injured volar plate were thicker in all the three types.

**Fig. 5:**
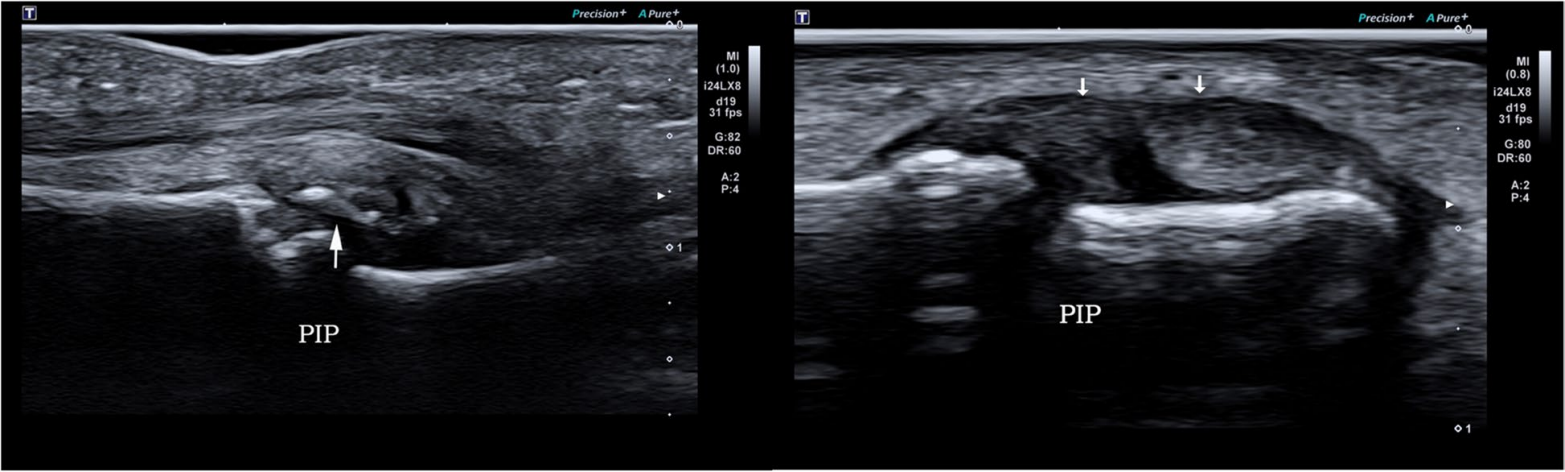
Volar plate injury accompanied by lateral collateral ligament injury. High frequency ultrasonography image of type A volar plate injury of a 33-year-old male accompanied by lateral collateral ligament injury. The arrow in the left figure indicates the avulsion bony fragment of the middle phalangeal base, and the arrow in the right figure indicates the disruption of the lateral collateral ligament. PIP: proximal interphalangeal joint

Ultrasound showed that among 41 patients with volar plate injury, 15 patients were accompanied by lateral collateral ligament injury (Fig. 5), including 3 cases of avulsion fracture of proximal attachment of lateral collateral ligament, 5 cases of lateral collateral ligament rupture and 7 cases of lateral collateral ligament tear.

**Fig. 6:**
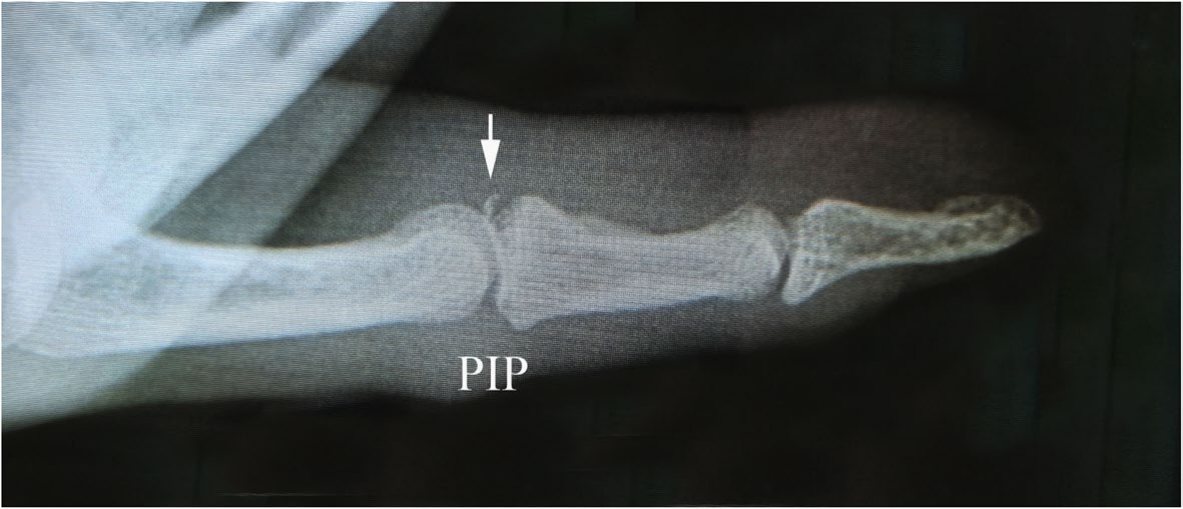
The X-ray findings of the same patient in Fig. 2 with type A volar plate injury. The arrow shows the avulsion bony fragment of the middle phalangeal base. PIP: proximal interphalangeal joint

For assessing avulsion fracture of the middle phalangeal base, most findings of the X-ray findings were in line with the ultrasonography results in type A (Fig. 6). X-ray revealed 13 cases of avulsion fracture at the middle phalangeal base in 41 patients. Among 13 cases of avulsion fracture, 3 cases were complicated with avulsion fracture at the proximal attachment of lateral collateral ligament.

For treatment, 8 out of 41 patients (4 type A, 4 type B) underwent open surgical repair either because in some cases the avulsion fracture was large which will influence PIP function, or because in some patients accompanied by lateral collateral ligament avulsion fracture or rupture, which according to our clinical experience that early surgical treatment is more beneficial. Moreover, the intraoperative clinical findings of those 8 cases were in good accord with preoperative ultrasonography examination results. The rest patients adopted conservation treatments with a dorsal blocking splint fixation of the PIP at 15° ~ 20° for about 3 weeks in type A and type B, and with a Bedford splint/buddy strapping and early active mobilization in type C. Follow-up of the patients confirmed a good outcome with satisfying results in both surgically and conservatively treated cases.

## Discussion

Acute closed volar plate injury is one of the common hand injury caused by finger hyperextension or rotation, often combined with lateral collateral ligament injury. Volar plate injury could cause pain, swelling, tenderness and instability of joints. Early diagnosis and treatment of volar plate injury is very important for its functional recovery. If the treatment is delayed, false button deformity or swan neck deformity of the PIP may occur, resulting in flexion contracture of the joint and loss of its function [4, 7].

In the past, the imaging examination of volar plate injury was rare. As is known to all, X-ray can show avulsion fracture directly [8]. But in full thickness tear of volar plate without fracture (type B) or partial thickness tear of volar plate (type C) X-ray shows no positive finding, which places restrictions on the use of X-ray in volar plate injury. With the increasing resolution of high-frequency ultrasound, its application has been extensively broadened in the diagnosis of musculoskeletal nervous system diseases [9, 10]. The results proved that high frequency ultrasound can clearly diagnose volar plate injury with high reliability and accuracy which could provide guidance as to choice of treatment options. The the inter-rater reliability for the classifications was high between two radiologists (κ = 0.963). High frequency ultrasound could clearly demonstrate the disruption of the volar plate with retraction of the proximal volar plate stump without fracture fragment in type B; in type C, the volar plate was hypoechoic and thicker which were still integrate shown by high frequency ultrasound. In type A, although high frequency ultrasound was inferior to X-ray in demonstrating the overall morphology of PIP, ultrasonography was as accurate and sensitive as X-ray in showing avulsion fracture fragment. Furthermore, compared to X-ray, ultrasonography allowed evaluation of real-time movement of the volar plate during active or passive movements of the PIP. More importantly, as everyone knows, ultrasound was a non-radioactive imaging method compared to X-ray [11, 12]. Therefore, we can use ultrasound to observe the change of thickness of the injured volar plate in the following-up after treatment [13]. As is well known, magnetic resonance imaging is also one of the effective methods for evaluating volar plate injuries. MRI showed the volar plate as clearly as ultrasonography and most MRI findings of volar plate injury were in line with that of the ultrasonography. The thickness of injured volar plates will gradually decrease with the reduction of swelling over time.

Regarding treatment of acute closed volar plate injury, clinical physicians recommend either conservative or surgical treatment based on the type and extent of the injury. Surgery should be adopted either because in some cases the avulsion fracture was large which will influence PIP function, or because in some patients accompanied by lateral collateral ligament rupture, which according to our clinical experience that early surgical treatment is more beneficial. For other conditions, conservative treatment would be more appropriate with a dorsal blocking splint fixation of the PIP at 15° ~ 20° for about 3 weeks in type A and type B, and with a Bedford splint/buddy strapping and early active mobilization in type C. Satisfactory outcome was confirmed in this study during clinical follow-up in both surgically and conservatively treated cases [14–16].

This study did have some limitations. First, an unavoidable problem is operator dependence, which requires ultrasound doctors to strengthen the knowledge of hand anatomy and improve the understanding of the disease. Second, the vast majority of volar plate injuries in this group were cured by conservative treatment, resulting in the lack of surgical confirmation of the original classifications. Third, all imaging cases of volar plate injury in this study have only undergone ultrasound and X-ray examinations without objective good standard such as high resolution 7T MRI, so the findings of the study can not easily expanded to clinical practice.

In conclusion, high frequency ultrasound can be served as a reliable, accurate, convenient and non-radioactive diagnostic imaging technique in the evaluation of both bony and simple volar plate injury. What’s more, real-time ultrasonography can help with assessing the function of volar plate.Ultrasound can provide a beneficial guidance to clinical decision-making and appropriate treatment adopting through accurate US classification. Further research is still needed to improve its clinical application in diagnosis and treatment of volar plate injury.

## Data Availability

The datasets used or analysed during the current study are available from the corresponding author on reasonable request.
